# Coexistence of meningioma and craniofacial fibrous dysplasia: a case series of clinicopathological study and literature review

**DOI:** 10.1186/s13023-024-03032-0

**Published:** 2024-01-30

**Authors:** Xiaowen Song, Zhi Li

**Affiliations:** 1 Department of Radiology, Center of Interventional Radiology and Vascular Surgery, Zhongda Hospital, Medical School, Southeast University, Nanjing, 210009 Jiangsu Province China; 2 Basic Medicine Research and Innovation Center of Ministry of Education, Zhongda Hospital, Southeast University, Nanjing, 210009 Jiangsu Province China; 3 Department of Neurosurgery, Beijing Tiantan Hospital, Capital Medical University, Beijing, 100070 China

**Keywords:** Meningioma, Craniofacial fibrous dysplasia, Clinicopathologic features, Differential diagnosis

## Abstract

**Background:**

The co-existence of meningioma and craniofacial fibrous dysplasia (CFD) is rare. Due to the similar radiological characteristics, it is challenging to differentiate such co-existence from solitary hyperostotic meningioma resulting in a dilemma of prompt diagnosis and appropriate intervention.

**Method:**

We conducted a retrospective review of the data from 21 patients with concomitant meningioma and CFD who were treated at Beijing Tiantan Hospital from 2003 to 2021. We summarized their clinicopathological features and performed a comprehensive literature review. Additionally, we tested the characteristic pathogenic variants in exon 8 and 9 of *GNAS* gene and the expression of corresponding α-subunit of the stimulatory G protein (Gα_s_) related to CFD to explore the potential interactions between these two diseases.

**Results:**

The cohort comprised 4 men and 17 women (mean age, 45.14 years). CFD most commonly involved the sphenoid bone (n = 10) and meningiomas were predominantly located at the skull base (n = 12). Surgical treatment was performed in 4 CFD lesions and 14 meningiomas. Simpson grade I-II resection was achieved in 12 out of the 14 resected meningiomas and almost all of them were classified as WHO I grade (n = 13). The mean follow-up duration was 56.89 months and recurrence was noticed in 2 cases. Genetic study was conducted in 7 tumor specimens and immunohistochemistry was accomplished in 8 samples showing that though *GNAS* variant was not detected, Gα_s_ protein were positively expressed in different degrees.

**Conclusions:**

We presented an uncommon case series of co-diagnosed meningioma and CFD and provided a detailed description of its clinicopathological features, treatment strategy and prognosis. Although a definite causative relationship had not been established, possible genetic or environmental interplay between these two diseases could not be excluded. It was challenging to initiate prompt diagnosis and appropriate treatment for concomitant meningioma and CFD because of its similar radiological manifestations to meningioma with reactive hyperostosis. Personalized and multi-disciplinary management strategies should be adopted for the co-existence of meningioma and CFD.

**Supplementary Information:**

The online version contains supplementary material available at 10.1186/s13023-024-03032-0.

## Background

Meningiomas, primarily arising from meningothelial arachnoid cells, are the most common intracranial tumors at present, accounting for almost one third of all primary central nervous system tumors [1]. Its incidence rate varies from 1.28 to 8.81 per 100,000 persons in different studies around the world [2, 3]. Fibrous dysplasia (FD) is an uncommon mosaic disorder resulting in replacement of normal bone with fibro-osseous tissue. The actual incidence of FD is once reported to be 10–30 in 1,000,000 persons, representing as many as 7% of benign bone tumors [4, 5]. It may occur in one single bone (monostotic FD), in multiple bones (polyostotic FD) or in combination with extra-skeletal disease. Craniofacial bones are the most common location affecting as many as 87% of patients with polyostotic FD [6–9].

The co-existence of meningiomas and craniofacial fibrous dysplasia (CFD) is a fairly uncommon condition which has only been described in a few case reports [10–16]. However, the clinical and radiological characteristics of this condition have not been well-demonstrated and the actual interactions between these two entities still remain unclear. Sporadic activating variants in the *GNAS* locus not only result in replacement of normal bone with fibro-osseous tissue in CFD lesions [4], but also is mutationally activated in various cancer types, such as growth hormone-secreting pituitary tumors, pancreatic cancer and colorectal cancer [17, 18]. It remains highly concerned whether *GNAS* gene is the common genetic predisposition between CFD and meningiomas.

Craniofacial FD typically demonstrates dense and sclerotic lesions or appears as an area of radiolucent ground glass matrix. Relevant differential diagnoses of CFD should consider meningiomas, Paget’s disease of the skull bone, and benign osteosclerotic lesions like osteoma [19]. Since meningioma itself could inflict the adjacent bones resulting in bone destruction with similar radiological manifestations to CFD [5, 20], differential diagnosis between bone-invasive meningiomas and concomitant meningiomas and CFD is clinically problematic.

This article was designed to describe a seldom seen series of coexisting meningiomas and CFD, demonstrate their clinical characteristics, explore the underlying interactions and pathogenesis, and discuss the difference between concomitant meningiomas and CFD and single hyperostotic meningiomas in order to facilitate diagnosis and improve treatment.

## Methods

### Patients’ selection

In the period 2003–2021, a total of 1176 patients diagnosed with CFD at Beijing Tiantan Hospital were retrospectively screened. The study finally enrolled 21 cases that were reported to have concomitant CFD and cerebral meningiomas. All patients underwent computed tomography (CT) and magnetic resonance imaging (MRI) for diagnosis and evaluation. CFD and meningiomas were diagnosed in accordance with histological examinations in patients managed with surgery, while for patients received conservative treatment, diagnoses were made according to typical radiological characteristics. Demographic characteristics, clinical manifestations, radiological and pathological features, treatment procedures and outcomes were recorded.

Follow-up was accomplished via telephone or at the clinic. CT and MRI was carefully evaluated and whether there was disease recurrence or progression was recorded.

This study was approved by the Institutional Review Board. Due to the retrospective nature of our study, the board waived the need for written consent.

### Immunohistochemistry and genetic analysis

Immunohistochemistry was performed to detect α-subunit of the stimulatory G-protein (Gα_s_) protein expression in meningioma specimens. The tissue sections were incubated with primary Gα_s_ antibody (1:100, sc-365855, Santa Cruz). Each stained slide was individually reviewed and independently scored by two neuropathologists. Genomic DNA was extracted from paraffin-embedded meningioma specimens using the Wizard Genomic DNA Purification kit following the manufacturer’s instructions (Promega, Madison, WI). The Gα_s_ encoding exons 8 and 9 of *GNAS* were amplified by PCR and sequenced by conventional Sanger sequencing (BigDye Terminator Cycle Sequencing Ready reaction kit, Applied Biosystems).

### Literature review

In addition, we searched 3 medical database, PubMed, EMBASE and Cochrane Library up to 2021 for published studies focusing on the coexistence of CFD and meningioma. The following combined terms ([MESH] “fibrous dysplasia” AND [MESH] “meningioma”) were used. A manual researching on the reference of identified studies was performed for more related studies.

## Results

### Clinical and radiological characteristics

**Fig. 1:**
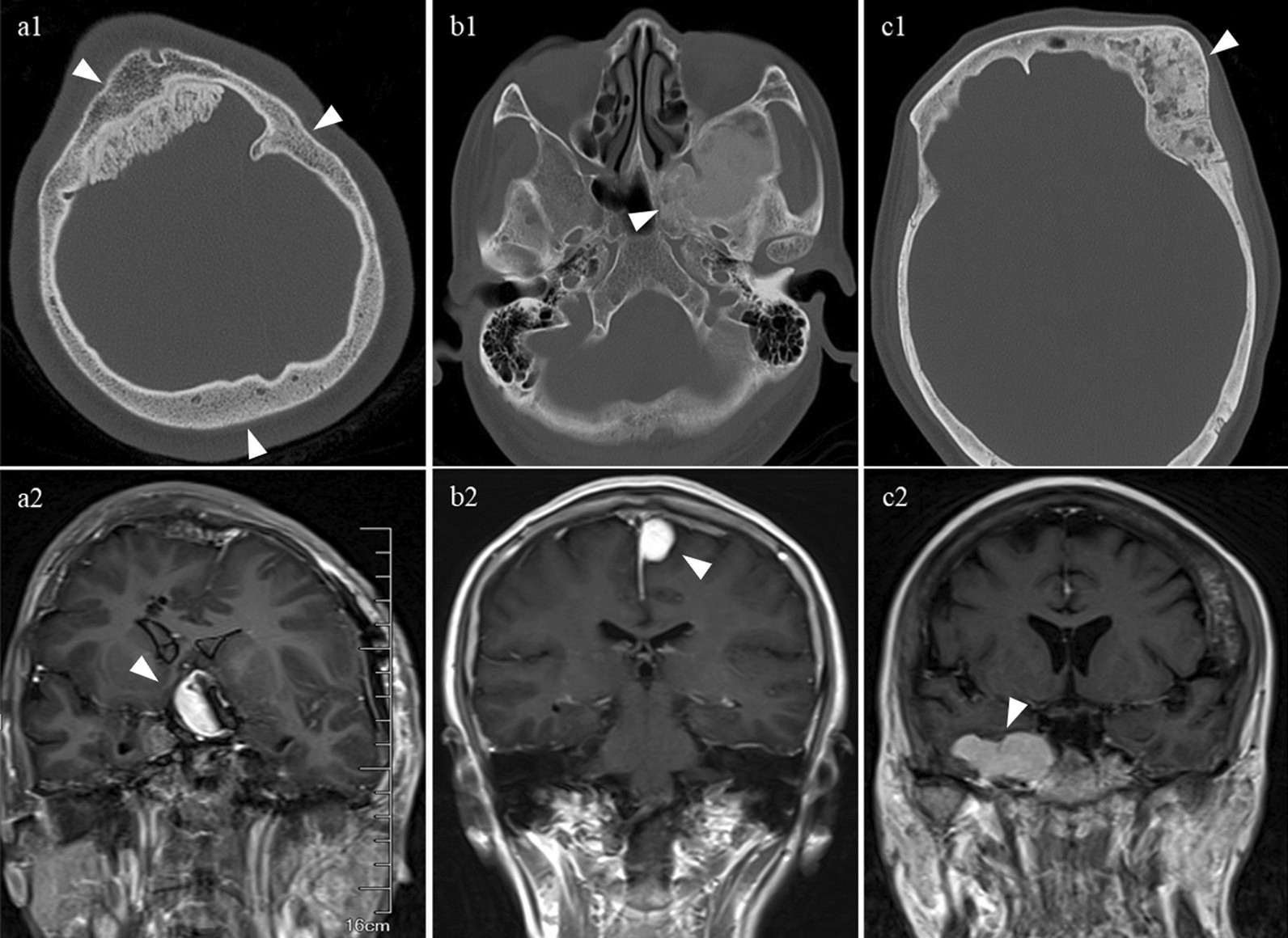
Radiological manifestations. **a1**, **a2** radiology of case 1 shows diffuse CFD in right maxilla, ethmoid sinus and sphenoid bone and tuberculum sellae meningioma; **b1–b2** radiology of case 19 shows CFD in left sphenoid bone and left frontal-parietal parafalx meningioma; **c1**, **c2** radiology of case 9 shows CFD in bilateral sphenoid, temporal and occipital bones and left frontal parasagittal meningioma

Among the 1176 CFD patients evaluated, concurrent meningiomas were found in 21 patients (17 females, mean age 45.14 years old). Only 1 patient was adolescent. The teenage boy had a heavy disease burden of CFD (Fig. 1a1) and a meningioma located at tuberculum sellae (Fig. 1a2). Tables 1 and 2 presented the distribution of CFD and meningiomas. The majority (57.14%) of meningiomas located at the skull base and most (47.62%) of the CFD lesions affected the sphenoid bone.

**Table 1:** Demographical, radiological, therapeutic and prognosis characteristics of the 21 patients

Variables	
Age (mean)	45.14 years old
Gender (%)	
Male	4 (19.05%)
Female	17 (80.95%)
Symptom	
Craniofacial deformity	2
Neurological dysfunction	8
Seizure	1
Headache/dizziness	7
Symptom free	4
Meningioma	
Location	
Convexity	2
Para-falx/para-sinus	4
Skull base	12
Ventricle	3
Treatment	
Resection	14
Gamma knife	1
Watchful waiting	6
Pathology	
Transitional	6
Mixed	1
Meningothelial	4
Fibrous	2
Metaplastic	1
Follow-up (mean)	
Lost	2
All patients	56.89 months
Surgical patients	56.64 months
Watchful waiting patients	57.60 months
Prognosis	
Recurrence after resection	2
Favorable outcome	19
Cranial fibrous dysplasia	
Location	
Sphenoid bone	10
Maxilla	2
Frontal bone	5
Temporal bone	5
Parietal bone	2
Occipital bone	2
Ethmoid sinus	2
Clivus	2
Treatment	
Surgery	4
Watchful waiting	17
Follow-up (mean)	
Lost	2
All patients	56.89 months
Surgical patients	47.00 months
Watchful waiting patients	59.53 months
Prognosis	
Progression	0
Favorable outcome	19

**Table 2:** Detailed clinical information of the 21 patients included in this study

No	Age /y	Gender	Onset symptom	Lesion location		Management		Simpson grade	Prognosis		
				CFD	Meningioma	CFD	Meningioma		Follow-up/ month	CFD	Meningioma
1	15	Male	Craniofacial deformity	Right maxilla, ethmoid sinus and sphenoid bone	Suprasellar region	Surgery	Surgery	III	80	Unchanged	No recurrence
2	31	Female	Headache	Sphenoid bone	Left parietal-occipital lobe (parafalx)	Watchful waiting	Surgery	I	118	Unchanged	No recurrence
3	39	Female	Hearing loss	Sphenoid bone	Right cerebellopontine angle	Watchful waiting	Surgery	I	57	Unchanged	Recurrence
4	47	Female	Vision loss	Right clivus	Right parasellar region	Watchful waiting	Surgery	II	65	Unchanged	No recurrence
5	54	Female	Vision loss and double vision	Left orbit and frontal bone	Right parasellar region	Watchful waiting	Surgery	I	120	Unchanged	No recurrence
6	58	Male	Seizure	Left maxilla	Left olfactory sulcus	Watchful waiting	Surgery	I	77	Unchanged	No recurrence
7	37	Male	Vision loss	Left frontal bone	Left sphenoid ridge	Surgery	Surgery	II	84	Unchanged	No recurrence
8	37	Female	Headache	Left orbit, frontal, sphenoid and temporal bones	Left tentorium and left frontal lobe (convexity)	Surgery	Surgery	I	12	Unchanged	No recurrence
9	36	Female	Symptom free	Bilateral sphenoid, temporal and occipital bones	Left frontal lobe (parasagittal)	Watchful waiting	Surgery	I	10	Unchanged	No recurrence
10	56	Female	Vision loss	Right temporal bone	Right anterior clinoid process	Watchful waiting	Surgery	I	118	Unchanged	No recurrence
11	61	Female	Dizziness	Left sphenoid	Left frontal lobe (parafalx)	Watchful waiting	Surgery	I	8	Unchanged	No recurrence
12	26	Male	Headache	Bilateral parietal bones	Right petroclival region	Watchful waiting	Surgery	III	24	Unchanged	Recurrence
13	66	Female	Headache	Left sphenoid bone	Posterior part of third ventricle	Watchful waiting	Surgery	II	8	Unchanged	No recurrence
14	51	Female	Double vision	Left frontal–temporal bones	Left cavernous sinus and sphenoid ridge	Surgery	Surgery	II	12	Unchanged	No recurrence
15	38	Female	Headache	Left sphenoid bone	Right lateral ventricle	Watchful waiting	Gamma Knife radiosurgery		24	Unchanged	No progression
16	18	Female	Craniofacial deformity and vision loss	Left frontal bone	Left parasellar region	Watchful waiting	Watchful waiting		72	Unchanged	No progression
17	54	Female	Hearing loss	Right clivus and temporal bone	Right frontal-parietal lobe (convexity)	Watchful waiting	Watchful waiting		36	Unchanged	No progression
18	60	Female	Dizziness	Sphenoid bone and sphenoid sinus	Left lateral ventricle	Watchful waiting	Watchful waiting		72	Unchanged	No progression
19	60	Female	Symptom free	Left sphenoid bone	Left frontal-parietal lobe (parafalx)	Watchful waiting	Watchful waiting		84	Unchanged	No progression
20	61	Female	Symptom free	Left ethmoid sinus	Right petroclival region	Watchful waiting	Watchful waiting		Lost		
21	43	Female	Symptom free	Right parietal bone	Right sphenoid ridge	Watchful waiting	Watchful waiting		Lost		

*CFD* cranial fibrous dysplasia, *NA* not available

Surgical intervention was executed in 14 meningiomas. The extent of resection was considered gross-total in 12 patients (Simpson grade I-II) and subtotal in 2 patients (Simpson grade III). No postoperative complications was noticed in the 14 surgically treated patients and none of them received any adjuvant therapy postoperatively. During the average 56.64-month follow-up after surgery, the radiological examinations showed 2 recurrences. Among the 7 unresected meningiomas, 6 opted for watchful waiting, while 1 was treated with Gamma Knife radiosurgery. For instance, case 19 was a 60-year-old female with CFD involving the left sphenoid bone led to the diagnosis of CFD (Fig. 1b1) and a parafalx meningioma at left frontal-parietal lobe (Fig. 1b2). The meningioma showed any sign of progression during the 84 months of “watchful waiting”. These unresected meningiomas showed no progression during the mean 57.60-month follow-up. Referring to the included 21 CFD lesions, only 4 were managed with operation. The mean follow-up time was 56.89 months, and no recurrence or progression was observed in any of the CFD lesions. For example, case 5 who were diagnosed with CFD involving the left frontal bone and left orbit (Fig. 1c1) and meningioma in the right parasellar region (Fig. 1c2). The unresected CFD lesion stayed stable during 120-month follow-up.

**Fig. 2:**
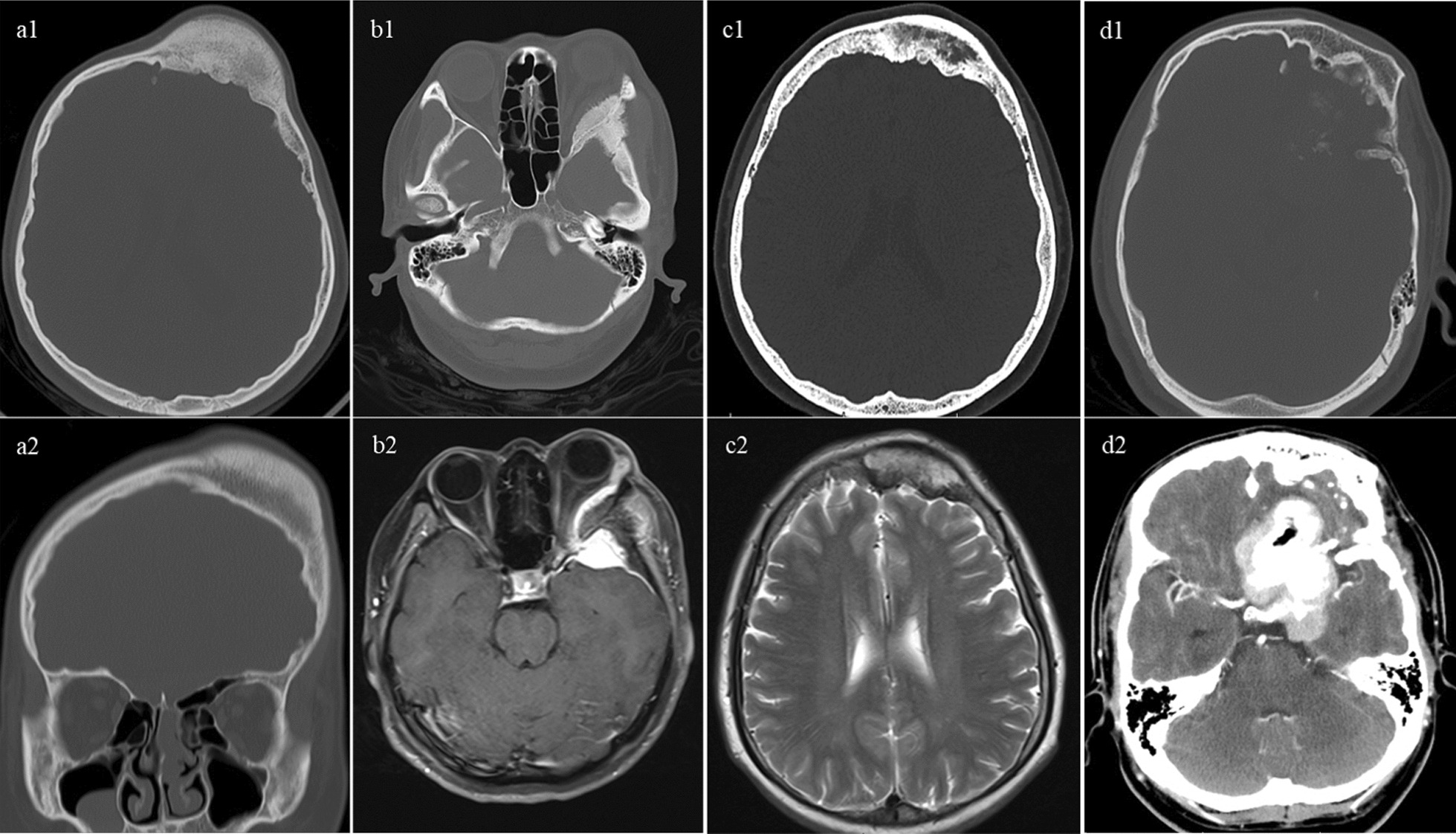
Differential diagnosis between bone invasive meningioma and concomitant meningioma and CFD. **a1, 2** typical CFD showing asymmetric expansive lesion at the left frontal bone with typical ground-glass matrix; **b1**, **b2** hyperostosis caused by meningioma revealing a sclerotic lesion of the left greater sphenoid wing with spiculated margins; **c1**, **c2** hyperostotic intraosseous meningioma with irregular inner table; **d1**, **d2** concomitant meningioma and CFD

CFD typically demonstrated dense, sclerotic lesions and was often associated with the term “ground glass bone matrix”. However, a smooth outer cortical contour always maintained (Fig. 2a). Although meningioma related hyperostosis (Fig. 2b) and intraosseous meningiomas (Fig. 2c) were also evaluated as sclerotic lesions, these lesions exhibited irregular and spiculated borders. Figure 2d demonstrated a co-existing meningioma adjacent to the CFD lesion, the involvement of the lamina interna cranii caused by the meningioma could complicate and interfere with the identification of CFD, making it challenging to differentiate the co-occurrence from the bone-invasive meningioma.

### Pathological characteristics and genetic results

**Fig. 3:**
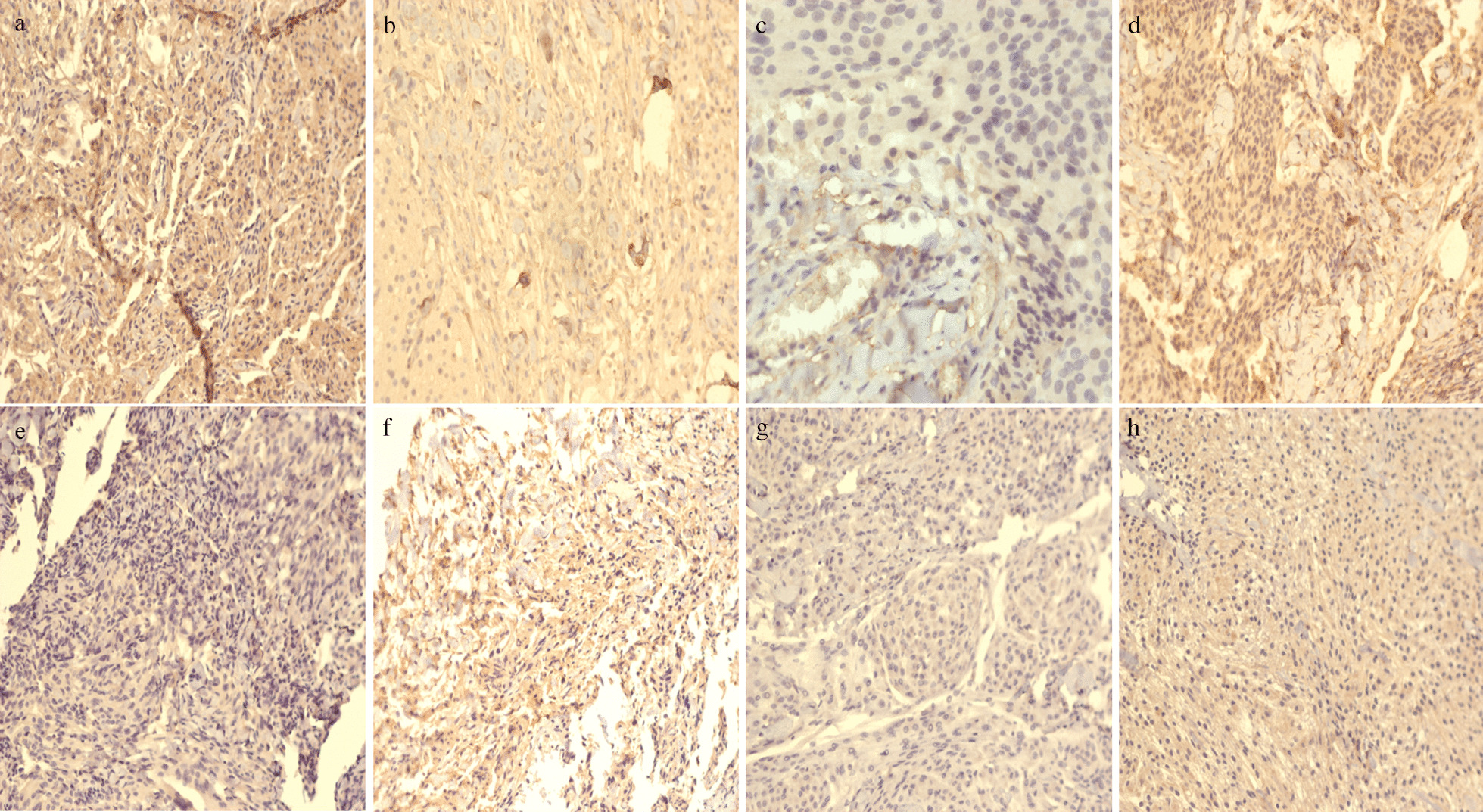
Immunohistochemical analysis of the Gα_s_ expression of the meningiomas in patients 1–8. Gα_s_ is strongly expressed (grade 2) in case 1 (**a**) and case 6 (**f**), moderately expressed (grade 1) in case 2–4 (**b**–**d**) and case 8 (**h**) and mildly expressed (grade 0) in case 5 (**e**) and case 7 (**g**)

Among the 14 surgically resected and pathologically examined meningiomas, transitional meningiomas were the most common type (6, 42.86%), and almost all the meningiomas (92.86%) were reported to be WHO I grade (Table 3) (Additional file 1). DNA sequencing was accomplished in 7 cases with no *GNAS* variant detected. In addition, 8 meningiomas were immunohistochemically examined and Gα_s_ expression was positive (grade 1 and grade 2) in 6 specimens (Fig. 3).

**Table 3:** Pathological characteristics of the resected meningioma specimens

No	WHO grade	Histological diagnosis	Gα_s_ protein	*GNAS* gene
1	WHO I	Transitional	2	Negative
2	WHO I	Mixed	1	Negative
3	WHO I	Meningothelial	1	Negative
4	WHO I	Meningothelial	1	Negative
5	WHO I	Fibrous	0	Negative
6	WHO I	Transitional	2	Negative
7	WHO I-II	Meningothelial	0	NA
8	WHO I	Transitional	1	Negative
9	WHO I	Metaplastic	NA	NA
10	WHO I	Meningothelial	NA	NA
11	WHO I	Transitional	NA	NA
12	WHO I	Transitional	NA	NA
13	WHO I	Fibrous	NA	NA
14	WHO I	Transitional	NA	NA

*NA* not available

Both of the meningioma samples with grade 2 Gα_s_ expression were transitional meningiomas (case 1 and case 6). Case 1 was a suprasellar meningioma co-existed with diffuse CFD involving right maxilla, ethmoid sinus and sphenoid bone and case 6 was a left olfactory groove meningioma concomitant with a CFD lesion inflicting left maxilla (Additional file 2). Although these 2 CFD lesions showing strongly positive Gα_s_ expression both involved maxilla, no definite correlation between the level of Gα_s_ expression and the location of CFD lesions could be drawn because of the limited sample size.

The two recurrent meningiomas (case 3 and case 12) were both WHO I grade. Case 3 was a meningothelial meningioma in the right cerebellopontine angle co-diagnosed with sphenoid bone CFD. Although without *GNAS* mutation, the meningioma revealed grade 1 Gα_s_ expression. Co-occurrence of CFD inflicting the parietal bone and a transitional meningioma in the right petroclival region was seen in case 12 (Additional file 2). Immunohistochemistry and genetic analysis was missing for this recurrent meningioma. No relation was found between recurrence and pathological characteristics.

### Literature review

Only 8 studies met the inclusion criteria and all of them were case reports. Detailed information of these 4 articles were described in Table 4. The mean age of the included patients was 29.38 years old with 6 males and only 2 females. Operations were reported in 5 meningiomas and 4 FD lesions. None of them explored *GNAS* variant and Gα_s_ expression in meningioma specimens and no hypothesis was put forward to explain the co-occurrence.

## Discussion

This article reported an infrequent series of concomitant meningiomas and CFD. To the best of our knowledge, this study was the largest case series highlighting the clinicopathologic features, treatment modalities and prognosis. In addition, we also reviewed the literature and discussed the challenges to differentiate such co-existence with solitary bone-invasive meningioma.

The actual mechanism of co-existed meningiomas and CFD remains unclear. There are several possible explanations: (1) genetic predisposition; (2) a purely coincidental event; (3) environmental influence as an irritating agent for the local proliferation and growth of the other [21–23]. Sporadic CFD is reported to be most common in children and adolescents and barely have any gender difference [24], which varies a lot from the age (mean age 45.14 years old) and gender profile (marked female: male ratio up to 4:1) of the included 21 CFD co-diagnosed with meningiomas. What’s more, the incidence rate of meningioma in the general population varies from 1.28 to 8.81 per 100,000 persons [2, 3, 25], however, the present study found 21 meningiomas in the 1176 CFD patients indicating much higher incidence of meningioma (1.8%). Therefore, it is reasonable to hypothesize a possible link between meningiomas and CFD. However, radiological imaging is more regularly performed in CFD patients increasing the chance of incidental findings of other intracranial lesions including meningiomas, which should also be taken into account when evaluating the actual mechanism of the co-existence. Additionally, previous case reports show quite different demographic and radiological characteristics. There were 8 cases included in the literature review. When compared with regular meningioma patients, their mean age was much younger (29.38 years old) and the female predominance was absent since male patients outnumbered female by a ratio of 3:1 [10–16, 26]. And inconsistent with the present findings showing only two meningiomas in the same side of CFD, most of meningiomas included in the previous reports were found to be adjacent to CFD lesions. Such random demographic and radiological profile also provides further evidence for the possibility that the co-existence might be coincidental.

FD is caused by a mosaic activating pathogenic variant in *GNAS* gene [27, 28], and the development of sporadic meningiomas also has genetic predisposition including *NF2, TRAF7, KLF4, AKT1* and *TERT* [29–31]. *GNAS* pathogenic variants have been previously found in various systems and has been reported to be associated with many extra-skeletal diseases such as thyroid hyperfunction, hormone-secreting pituitary tumors, pancreatic cancer, breast cancer and colorectal cancer [6–9, 17, 18, 32, 33].Furthermore, *GNAS* pathogenic variant is also detected in an endothelial meningioma with multiple recurrences recently [34]. However, the present results did not find any pathogenic *GNAS* variant in the 7 meningiomas analyzed, consistent with the study of Eun who examined 13 meningioma samples [35]. To date there is no evidence concerning the definite role of *GNAS* variants in the co-occurrence of meningioma and CFD. The current study also tested the Gα_s_ protein encoded by *GNAS*. Though there was no association between Gα_s_ expression and the histology of meningiomas, the different expression levels of Gα_s_ in the meningioma specimens delineated the possibility that the development of these two diseases might share a common molecular pathway. Case 1 showed a 15-year-old transitional meningioma with strong positive Gα_s_ expression. The relatively young age of meningioma onset and the multiple surgeries of CFD provided some evidence for the hypothesis that CFD might have environmental influence as an irritating agent on the occurrence and development of meningiomas.

Bone involvement is a major concern in meningioma [36], which is documented in 20–68% of meningiomas by histopathological studies [37] and is proved to influence tumor recurrence and prognosis [38]. Bone invasive meningiomas are associated with *NF2* and *TRAF7* variants [39]. Radiographic evidence of bone involvement includes hyperostosis, bone sclerosis and osteolytic lesions [40]. Both characterized by an increased bone density involving the craniofacial bones, meningioma associated hyperostosis and CFD can be confounded easily resulting in the dilemma to differentiate concomitant meningioma and CFD from meningioma with hyperostotic bone involvement. Seen in 25–49% of meningiomas [41], meningioma associated hyperostosis most frequently affects the convexity and sphenoid wing [5, 42] and is featured by irregular inner surface margins and diffuse “hairy spicules” trabecular hyperostosis without the destruction of trabecular structures [43–45]. Additionally, as a special condition of meningioma restricted in bone (accounting for about 2%) [46, 47], intraosseous meningiomas are readily evaluated as sclerotic lesions with irregular and spiculated borders [48–50]. However, CFD prototypically appears as an area of radiolucent homogeneous ground glass matrix with a smooth cortical contour [51–53]. Therefore, it can be inferred that the key to diagnose CFD is the regular contours of cortical table, but when the co-exist meningioma was adjacent to CFD, the intact lamina interna cranii could be destroyed, making the differential diagnosis more complicated.

Misdiagnosis may influence the treatment preferences and patients’ prognosis. The management strategy should be based on the accurate diagnosis. If it is considered to be meningioma with reactive hyperostosis or intraosseous meningioma, complete resection might be recommended to reduce recurrence and improve prognosis [39, 54]. However, if the patient is diagnosed with co-existed meningioma and CFD, “watchful waiting” treatment of the bone lesion may be acceptable especially when there is no CFD related symptom since FD turns to be stable after adolescence. This managemeng strategy is further proved by the current series of co-existed meningioma and CFD. Although only 4 CFD was surgically resected, most patients had favorable prognosis without any obvious CFD progression suggesting that the co-existence of CFD may not influence the prognosis of meningiomas. However, if important structures are compressed causing complaints, surgical resection should be considered. In addition, when meningioma is located in close juxtaposition of CFD, the bone lesions caused by CFD will make the exposure laborious for the resection of meningioma. In this situation, surgical resection of CFD can be recommended. However, whether these two diseases should be managed at one session ought to be evaluated carefully [55, 56]. Therefore, interdisciplinary and more personalized management should be adopted for patients diagnosed with concomitant CFD and meningioma.

Our study has some limitations. Firstly, due to the rarity, the sample size of qualified cases is limited. More cases are needed to strengthen the reliability. Secondly, no definite mechanism concerning the coexisting meningioma and CFD is clarified which still needs further exploration and verification. Technologies such as whole exome sequencing can be considered to study the common molecular pathway of meningioma and CFD in future researches.

**Table 4:** Literature review of co-existed meningiomas and CFD

Author	Publication year	Gender	Age/y	Onset symptoms	Type of CFD	Location		Treatment		Histological examination
						CFD	Meningioma	CFD	Meningioma	
Settecase et al. [16]	2016	Male	13	Enlarging lump on right forehead	MAS	Involving the entire skull	Multiple: right frontal and right posterior falx	Untreated	Untreated	NA
Alves et al. [15]	2009	Male	35	Growing mass	CFD	Frontal bone	Right frontal	Surgery	Surgery	Meningothelial
Ghosal et al. [13]	2007	Male	25	Diminishing vision and seizures	CFD	Sphenoid sinus and bone	Right frontal	Surgery	Surgery	Atypical lymphoplasmacyte-rich (WHO grade II)
Tasar et al. [14]	2004	Male	20	Exophthalmus and lost vision of right eye	CFD	Right frontal and temporal bones	Multiple: sphenoidal-temporoparietal	NA	NA	NA
Gao et al. [26]	2002	Female	37	Headache, dizziness and seizure	CFD	Right sphenoid bone	Right middle cranial fossa	Surgery	Surgery	Syncytial
Bayas et al. [57]	1999	Female	38	Myelopathy	MAS	Left maxilla, left ramus and right corpus mandibulae	T3-T4	Untreated	Surgery	Psammomatous
Fehlow et al. [58]	1992	Male	17	Psychic maldevelopment	MAS	Right frontal and temporal bones	Right temporal	NA	NA	NA
Frankel J, et al. [59]	1988	Male	50	Skull swelling and double vision	CFD	Frontal, parietal and sphenoid bones	Left parietal	Surgery	Surgery	Meningothelial (infiltrating bone)

*CFD* cranial fibrous dysplasia, *MAS* McCune-Albright syndrome, *NA* not available

## Conclusion

We reported a seldom seen case series of co-diagnosed meningioma and CFD and provided a detailed description of their clinicopathological features, treatment strategy and prognosis. Although a definite causative relationship is still undefined, possible genetic or environmental interplay between these two diseases cannot be excluded and requires further investigations. It can be quite intriguing to be differentiated from bone invasive meningiomas. The comprehensive assessment of this seldom seen and challenging condition in the present study can provide more profound understanding of this co-occurrence thus facilitating the diagnosis and helping with the determination of the appropriate treatment strategy.

### Supplementary Information

**Additional file 1: Fig. 1**. Representative images of meningioma pathology. (**a**) Pathological hematoxylin–eosin staining of case 1 tumor specimen indicating transitional meningioma (WHO I grade); (**b**) Pathological hematoxylin–eosin staining of case 5 tumor specimen showing fibrous meningioma (WHO I grade); (**c**) Pathological hematoxylin–eosin staining of case 9 tumor specimen reporting metaplastic meningioma with a Ki-67 label index of 3% (WHO I grade).

**Additional file 2**: Clinical descriptions and radiological presentations of 21 included cases.

## Data Availability

The datasets generated and/or analyzed during the current study are not publicly available due to individual privacy of the patients included but are available from the corresponding author on reasonable request.
