# Supplementary figures and images for: Coexistence of meningioma and craniofacial fibrous dysplasia: a case series of clinicopathological study and literature review
Source: Orphanet J Rare Dis. 2024 Jan 30;19:30. doi: 10.1186/s13023-024-03032-0 (PMC10826192; doi:10.1186/s13023-024-03032-0)

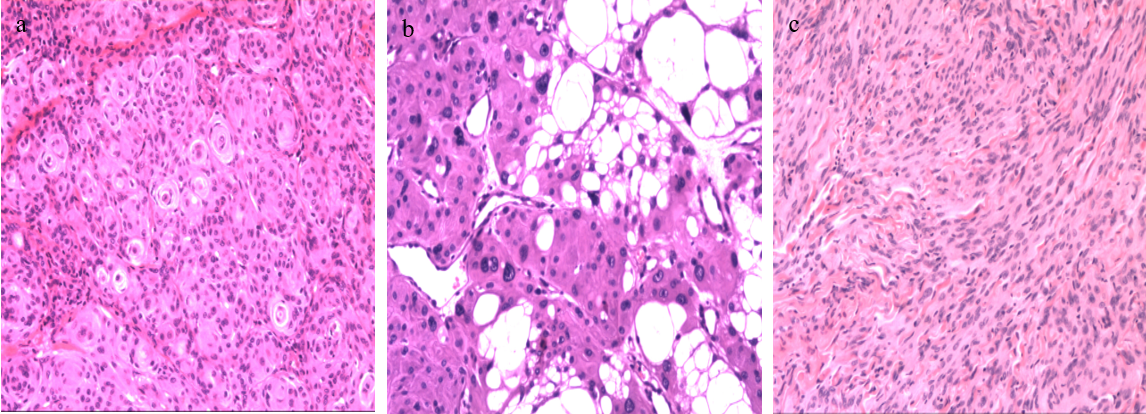

Supplement: Supplementary file 1 — Additional file 1: Fig. 1. Representative images of meningioma pathology. (a) Pathological hematoxylin–eosin staining of case 1 tumor specimen indicating transitional meningioma (WHO I grade); (b) Pathological hematoxylin–eosin staining of case 5 tumor specimen showing fibrous meningioma (WHO I grade); (c) Pathological hematoxylin–eosin staining of case 9 tumor specimen reporting metaplastic meningioma with a Ki-67 label index of 3% (WHO I grade). [file 13023_2024_3032_MOESM1_ESM.tif]
