# Supplementary material for: Coexistence of meningioma and craniofacial fibrous dysplasia: a case series of clinicopathological study and literature review
Source: Orphanet J Rare Dis. 2024 Jan 30;19:30. doi: 10.1186/s13023-024-03032-0 (PMC10826192; doi:10.1186/s13023-024-03032-0)

Case 1

This was a 15-year-old male. During his 15 years medical history of CFD, two plastic surgeries were performed for the facial asymmetry. An Intracranial space-occupying lesion had been found when regular follow-up checks were accomplished. And he was admitted into our center for the resection of the mass. On admission, the patient had asymmetrical craniofacial bones and [exophthalmos](http://www.baidu.com/link?url=RFFo2PofIiyfZN3z02YKNmr7ZKXml6-siwSZBM9oXkCuWCEU5YXh5cimw5La5GGfuvlKfZ3vTVvva2r1D8TetvBM8oUyfQtxhA2DRtuCDoS) because of CFD and also complained about vision loss and visual field defect resulted from the occupying lesion located at [Tuberculum sellae](https://www.baidu.com/link?url=YKm_OYGmI93U2C03epRR13vWMuREBVAZoes4lf_e_JDDIshdIwAyfzojpXINfbOSrk9Y_ASjxXkBaFRGEcQIAoFHs6Oxt_c5JyKhbhaRq-qpUwE7oZ45EXx04Ux75MZB&wd=&eqid=9a76d9e300166f09000000036273ccff). Scoliosis was diagnosed during pre-operative evaluations. Simpson grade III resection was executed through left [frontopterional craniotomy](http://www.baidu.com/link?url=9ahT8woWf7oj4MELsTEpHkNwZg0QdDFC4Ey3OtyY3o8OpkxvY4bfeHwKvD0LMbDr44zKFNxbk24IWMAyMS4AGK) and postoperative pathologic diagnosis was transitional meningioma (WHO I grade). There was no newly onset neurological deficit after surgery. And during the 80-month follow-up, no progression of CFD and no recurrence of meningioma were noticed.


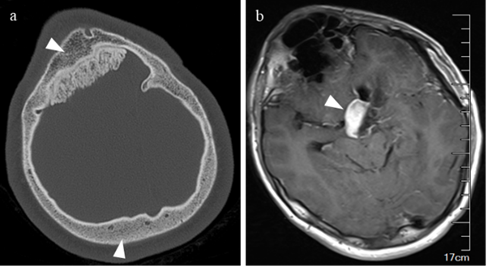


Case 2

This was a 31-year-old female. Head CT scan was performed when she went to the clinic and complained about intermittent headache. A parafalx meningioma at left parietal lobe and CFD of the sphenoid bone was found. The meningioma was removed completely (Simpson grade I) through left parietal-occipital approach while her CFD lesion was managed conservatively. Post-operative pathologic diagnosis was mixed meningioma (WHO I grade). During the 118 months follow-up, both of the two diseases remained stable.


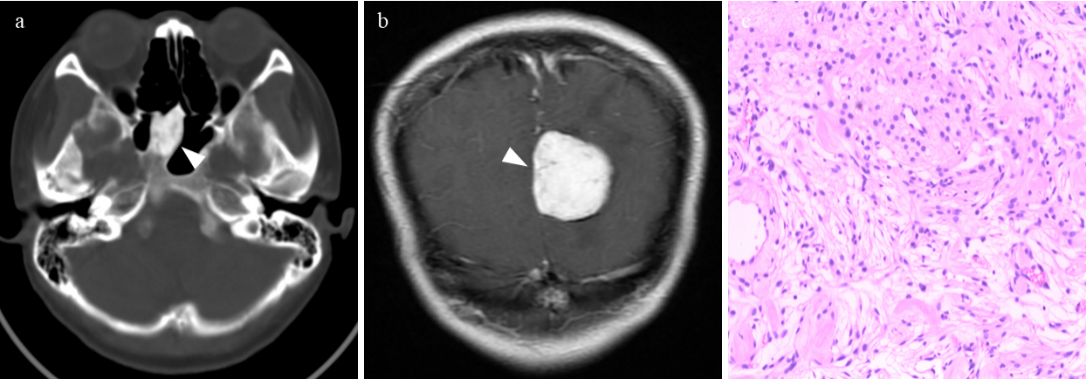


Case 3

The 39-year-old female was admitted into our center complaining about hearing loss and facial numbness on the right side for 3 years. Head CT and the MRI reported a cerebellopontine angle meningioma and CFD involving the sphenoid bone. The meningioma was resected completely (Simpson grade I) through a suboccipital retrosigmoid approach and the postoperative pathologic examination indicated meningothelial meningioma (WHO I grade). However, the meningioma was found recurrent after 57 months follow-up. Although the CFD lesion was left unresected, there was no sign of progression.


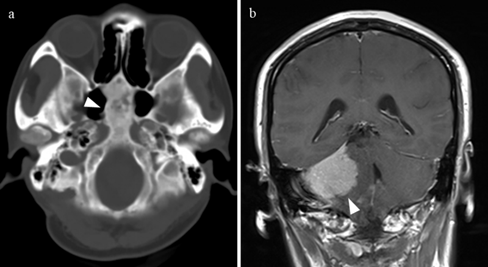


Case 4

The 47-year-old female had blurred vision for 3 months. MRI showed a right parasellar meningioma and CFD involving the clivus. Simpson grade II resection was performed through left [frontopterional craniotomy](http://www.baidu.com/link?url=9ahT8woWf7oj4MELsTEpHkNwZg0QdDFC4Ey3OtyY3o8OpkxvY4bfeHwKvD0LMbDr44zKFNxbk24IWMAyMS4AGK) and postoperative pathologic diagnosis was meningothelial meningioma (WHO I grade). CFD lesion was treated conservatively. Following 65 months follow-up, both lesions showed no sign of progression.


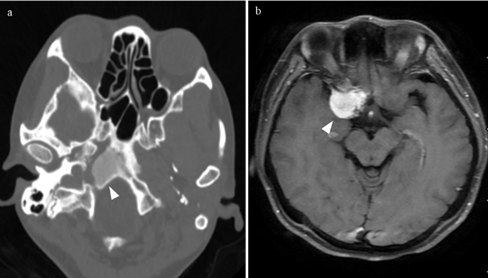


Case 5

The 54-year-old female was admitted into our center for “vision loss and double vision”. She had a 40 years medical history of CFD involving the left frontal bone and left orbit and no treatment was done. MRI showed a meningioma in the right parasellar region. Simpson I grade resection of the meningioma was fulfilled. She experienced no vision deterioration after surgery and postoperative pathologic examination indicated fibrous meningioma (WHO I grade). Both conditions stayed stable during 120-month follow-up.


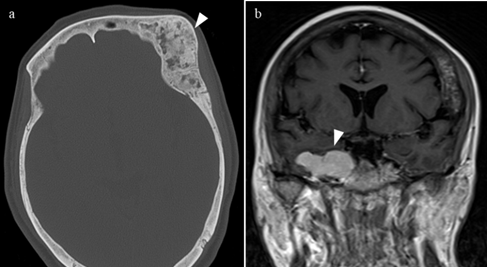


Case 6

The 58-year-old male was found to have a olfactory groove meningioma when MRI was assigned due to his seizure. On admission, craniofacial asymmetry was noticed and the patient reported that biopsy was done for the lesion in his maxilla indicating CFD. The meningioma was completely removed through left frontolateral approach. During 77 months follow-up, no progression was found for both lesions.


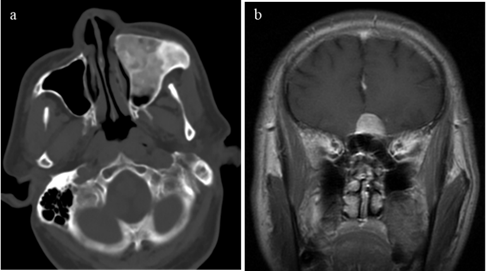


Case 7

The 37-year-old female was diagnosed with CFD 28 years ago for the lesion implicating his left frontal bone. The patient underwent meningioma resection 10 years ago. He was admitted into our center because of vision loss for 2 years. Head CT indicated a recurrent left sphenoid ridge meningioma. After Simpson grade II resection, the lesion was reported to be a meningothelial meningioma (WHO I-II grade). Both entities showed stayed stable after being followed up for 84 months.


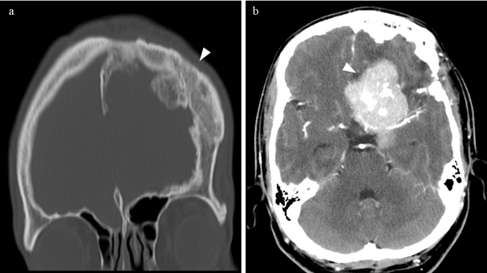


Case 8

This was a 37-year-old female. Head CT was done for vision loss. Diffuse CFD involving orbit, frontal, sphenoid and left temporal bones and multiple meningiomas at left frontal lobe and tentorium were found. Gross total resection of the meningioma was accomplished and was reported to be transitional meningioma. When removing the meningioma, CFD lesion involving the left frontal bone and left orbit was resected and reconstructed with titanium plate at the same time. The other parts of CFD were left untreated. After being followed up for 12 years, both entities remained unchanged.


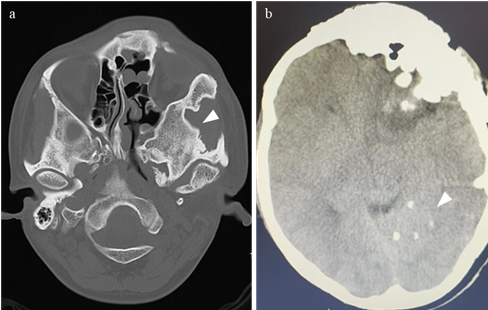


Case 9

This 36-year-old female was diagnosed with CFD 19 years ago according to the painless swelling of the sphenoid, temporal and occipital bones while no treatment was done. She was found to have a parasagittal meningioma at left frontal lobe during regular physical examinations. ~~She~~ After gross-total resection, pathological examination of the meningioma reported metaplastic meningioma with a Ki-67 label index of 3% (WHO I grade). During 10-month follow up, the radiological examinations detected no progression of CFD and no recurrence of meningioma.


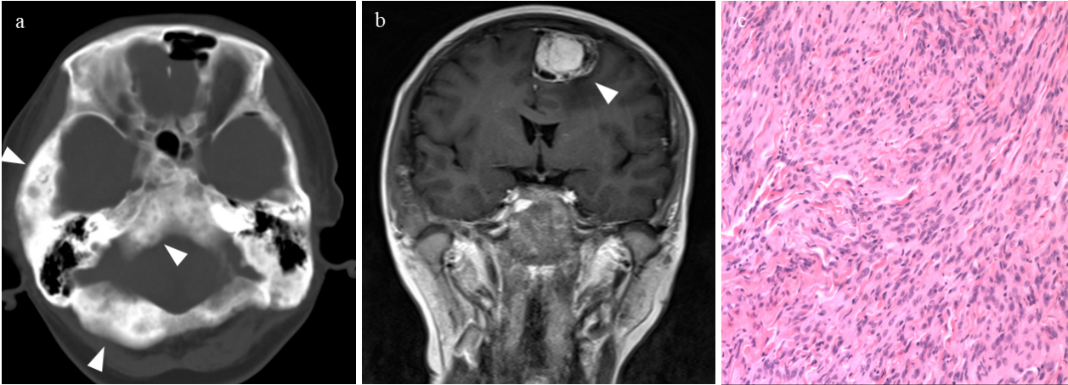


Case 10

This patient was a 56-year-old female. Head CT was done because she had a severe headache and right vision loss for almost 1 year. The radiological examination found a right parasellar tumor and CFD lesion implicating right temporal bone. The occupying lesion was completely resected (Simpson grade I) through subfrontal approach, which was proved to be a meningothelial meningioma (WHO I grade). After a 118-month follow-up, both abnormlities remained stable though the CFD was managed conservatively.


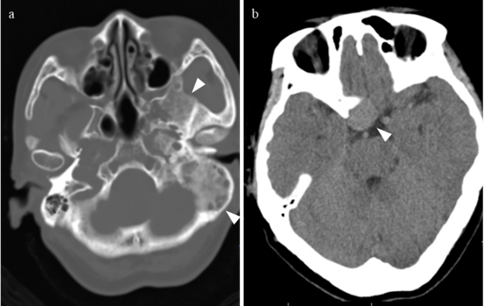


Case 11

The 61-year-ole female patient went to the clinic for dizziness. MRI was done and found a parafalx meningioma at left frontal lobe. Simpson grade I resection was accomplished through a coronal incision and the lesion was confirmed to be a transitional meningioma (WHO I grade) by the pathologic examination. The post-operative CT reported a classic left sphenoid CFD lesion. Following the 8 months follow up, neither of the abnormalities showed any sign of progression.


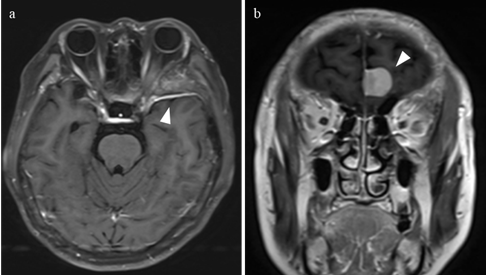


Case 12

This case was a 26-year-old male diagnosed with a right petroclival meningioma and CFD of the parietal bones according to the typical radiological manifestation. His meningioma was partially removed (Simpson grade III) through the subtemporal-anterior transpetrosal approach which was reported to be transitional (WHO I grade). After 24 months, the patient went back to our center for regular post-operative follow-up, the residual meningioma showed obvious progression though the CFD remained stable.


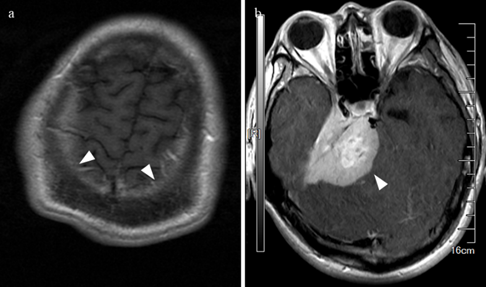


Case 13

This 66-year-old female patient complained about unstable walking for 3 months and an occupying lesion was showed in the posterior part of the third ventricle. Her MRI also reported a CFD lesion implicating the left sphenoid bone. Left suboccipital transtentorial approach was adopted to remove the occupying lesion (Simpson grade II), which was proved to be a fibrous meningioma (WHO I grade). The sphenoid FD was managed conservatively. There was no sign of recurrence or progression after being followed up for 8 months.


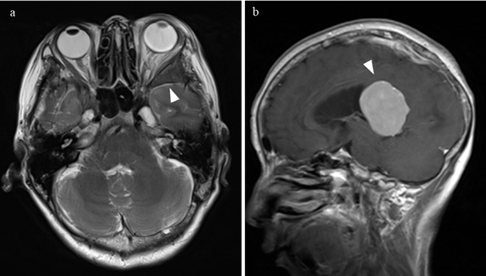


Case 14

The 51-year-old female was admitted into our center for double vision. Facial asymmetry and left exophthalmos was noticed. Meningioma located in the left cavernous sinus and sphenoid ridge. Diffuse CFD of left orbit and frontal-temporal bones was diagnosed according to the classic radiological manifestation showed in the head CT. The meningioma was removed (Simpson grade II) and the CFD lesion was partially resected. One-stage plasty with titanium plate was executed as well. Post-operative pathology confirmed the radiological diagnosis. Both diseases remained stable after 12-month follow-up.


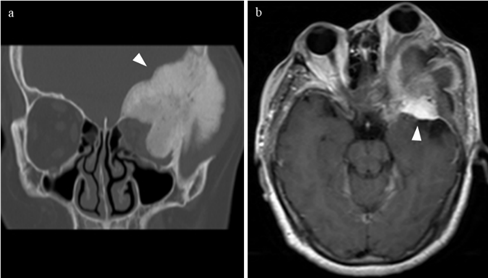


Case 15

This case was a 38-year-old female. MRI was done for her intermittent headache. The meningioma found in the right lateral ventricle was managed with Gamma knife while the left sphenoid FD was left untreated. No sign of progression was showed during the 24-month follow-up.


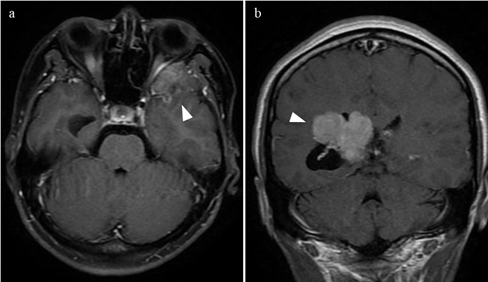


Case 16

The 18-year-old female went to the clinic for craniofacial deformity. Her head CT reported diffuse CFD involving left frontal bones and a meningioma in the left parasellar region. Regular clinic follow up lasted 6 years and no progression was shown.


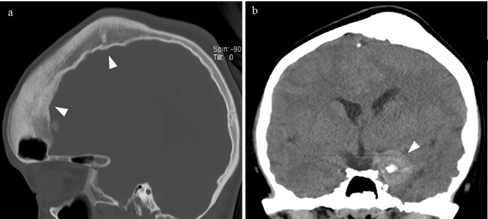


Case 17

This case was a 54-year-old patient. Artificial stapes were implanted for her hearing loss. During her pre-operative evaluation, head CT and MRI were done. CFD of the left clivus and temporal bone as well as a convexity meningioma in the left parietal lobe was found. After 36 months, the lesions remained stable though without surgery.


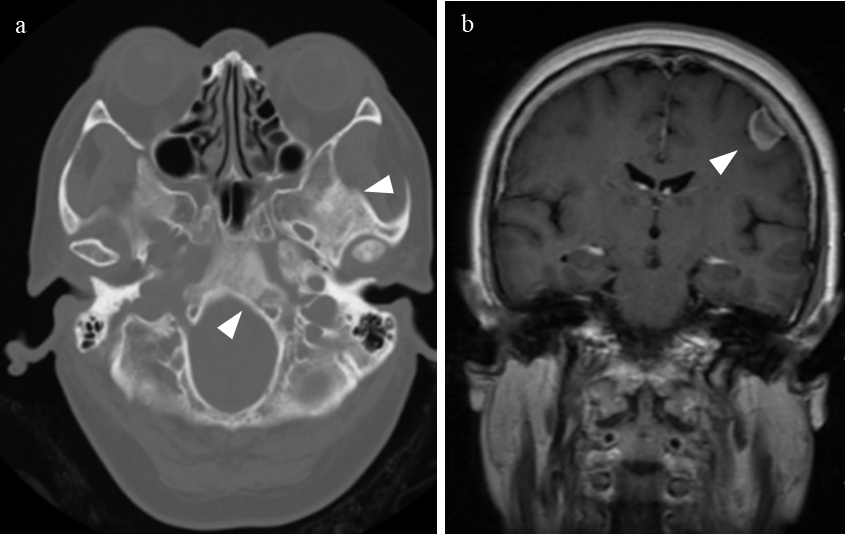


Case 18

This was a 60-year-old female. She came to the clinic for dizziness. Her MRI indicated sphenoid FD and a meningioma in the left lateral ventricle. There was no change of the two lesions during 72-month follow-up.


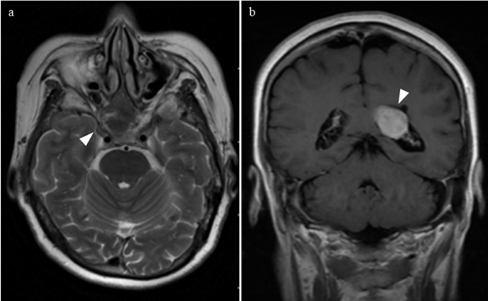


Case 19

This was a 60-year-old female. A typical “ground glass” manifestation involving the left sphenoid bone led to the diagnosis of CFD. Additionally, a parafalx meningioma at left frontal-parietal lobe was found during regular physical examinations. The patient did not receive surgical treatment for these two diseases for there was no related symptom. During the 84 months of “watchful waiting”, neither of these conditions showed any sign of progression.


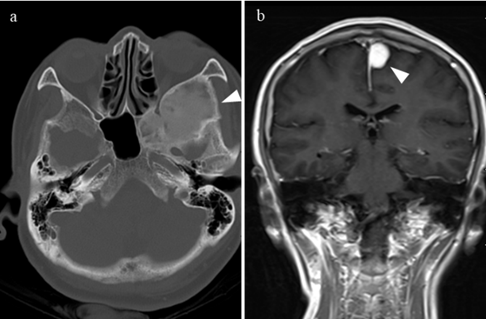


Case 20

The 61-year-old female went to the clinic for regular physical examination. CFD of the left ethmoid sinus and a right petroclival meningioma was reported. Watchful waiting was advised. However, the patient did not come back for outpatient follow-up.


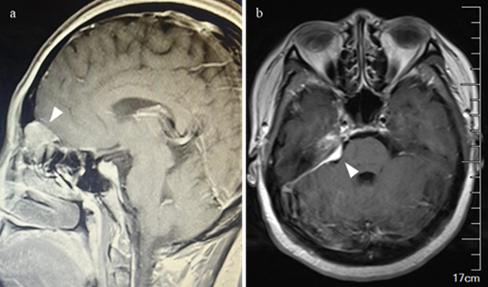


Case 21

This was a 43-year-old female. MRI was done for her regular physical examination. CFD of right parietal bone was found as well as a right sphenoid ridge meningioma. The patient was unwilling to have surgery and lost follow-up.


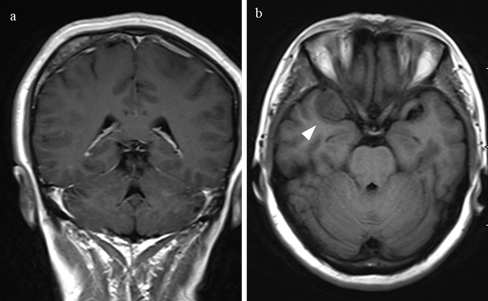

Supplement: Supplementary file 2 — Additional file 2: Clinical descriptions and radiological presentations of 21 included cases. [file 13023_2024_3032_MOESM2_ESM.docx]
